# Hypertension due to financial mismanagement

**DOI:** 10.3389/fpubh.2026.1855264

**Published:** 2026-07-15

**Authors:** Muhammad Akram, M. R. Muhammad Ishaque, Ho Soonmin, Md. Iqbal Bahar Chowdhury

**Affiliations:** 1 Department of Eastern Medicine, Government College University Faisalabad, Faisalabad, Pakistan; 2 Department of Eastern Medicine, University of Sindh, Jamshoro, Sindh, Pakistan; 3 Faculty of Health and Life Sciences, INTI International University, Putra Nilai, Negeri Sembilan, Malaysia; 4 School of Science and Engineering, United International University, Dhaka, Bangladesh

**Keywords:** cardiovascular risk, economic instability, financial mismanagement, financial stress, hypertension, mental health

## Introduction

Hypertension is an ongoing medical issue where blood pressures will always be higher than normal which puts people at risk of having a heart attack, stroke or kidney failure (1). All of us know the risk factors for developing hypertension, which include genetics, poor diet and lack of exercise; but lately there has been an increase in knowledge about the effect of psychosocial factors on developing and managing hypertension (such as stress and anxiety from financial mismanagement). Financial mismanagement is when an individual cannot manage their income, expenses, savings and debt and becomes overwhelmed by the stresses and anxiety produced from mismanaging money (2). In a fast-paced, economically challenging world, many people find themselves under ever-increasing pressure from unplanned expenses, and unexpected events, and will experience mental health issues and emotions related to this financial pressure and subsequently will develop or worsen their hypertension (3).

The current generation of youth is experiencing financial pressure like never before due to increased cost of living, high debt burdens, and increased social pressure via social media. Given that the cost of houses and inflation rates have exceeded income levels, most young people have been experiencing financial pressure and find it hard to secure their future financially. Inflation in homes, food, and medical bills has left most young people struggling from paycheck to paycheck. The constant connectivity of digital platforms makes young people compare themselves constantly, with social media being named as the cause of expensive purchases.

## Data and discussion

According to research, chronic stress and high blood pressure are highly correlated. Financial mismanagement is one of the major factors contributing to chronic stress by producing ongoing uncertainty and insecurity in a person's life or situation. When individuals have debt, unpaid bills, or don't have enough savings, they may feel anxious, have sleep problems, and experience emotional distress. The body's response to these psychological stressors triggers the stress response system and causes the secretion of hormones, such as cortisol and adrenaline (4). Continuous secretion of these hormones causes the heart rate to increase, results in blood vessels becoming smaller, and can cause long-term hypertension (high blood pressure).

Financial problems really do affect blood pressure. When you are worried about money all the time your body gets stressed. That makes your blood pressure go up. This can happen fast. If you are worried about money for a time you might get high blood pressure that does not go away. That is not good because it can cause strokes. Financial problems and strokes are connected in a way. If you are stressed about money all the time that can cause problems that lead to strokes. If you have a stroke, you will probably have to pay a lot of money for medical bills and you might not be able to work, which makes your financial problems even worse. When people are having a lot of money trouble, they might start doing things that're not good for them like eating badly, smoking or drinking too much. All those things can make you more likely to have a stroke.

Financial problems can make you more likely to get heart disease. When you are stressed about money for a time that can really hurt your heart. You might even have a heart attack. Financial problems can make your body change in ways like gaining weight or getting sick more easily. They can also make you do things that're not good for you every day. When you are stressed about money your brain sends out stress hormones like cortisol. Adrenaline all the time. Those hormones can make your blood pressure and heart rate go up which is not good for your heart. Financial problems can really hurt your heart. Make you get heart disease.

In addition, financial stress often leads people to make poor choices in terms of eating and drinking. People experiencing economic pressures are likely to consume unhealthy foods due to their low cost and high sodium content and have an increased risk of developing high blood pressure. Furthermore, behaviors associated with stress, such as smoking, drinking, and not exercising, contribute to an increased risk for developing high blood pressure. In addition to these things, limited financial means can also prevent someone from obtaining health care services, medications, or regular visits to see a physician for early diagnosis and treatment of high blood pressure (5). Studies conducted on different socioeconomic groups indicate that people with less financial security have a significantly greater prevalence of hypertension than those with greater financial security. Individuals who experience job insecurity and live from paycheck to paycheck have elevated levels of stress hormones and increased risks for cardiovascular disease. Furthermore, these findings present an opportunity to add financial health to the definition of health.

### Physiological mechanisms linking financial stress to hypertension

Financial difficulties trigger a long-term response from the body's fight or flight system (HPA-axis and sympathetic system) when there is continual uncertainty regarding finances (6). When someone is experiencing continued uncertainty regarding financial situations, the body will produce and release stress hormones such as cortisol (CORT) and epinephrine. As a result, CORT and epinephrine are released, which leads to an increase in heart rate; the body's blood vessels will constrict; and blood pressure will increase. Prolonged activation of this system will eventually result in both structural and functional changes within the blood vessels (e.g., decreased elasticity and increased peripheral resistance), causing long-term high blood pressure (hypertension). Long term exposure to stress hormones also leads to an increased level of inflammatory factors and an impairment to the endothelial layer of the vasculature, which continues to elevate cardiovascular risk (7).

### Behavioral risk factors associated with financial mismanagement

Poor financial management causes maladaptive coping techniques to develop, which do so indirectly and thus increase the likelihood of hypertension. Individuals experiencing financial stress use unhealthy eating habits (for example, eating less expensive, high sodium and fatty foods) as a form of coping. This type of dietary pattern has been shown to increase blood pressure. Further, individuals who are experiencing stress because of their financial situation often exhibit behaviors that are consequences of this stress (e.g., smoking, drinking excessive coffee, consuming excessive alcohol). This means that these behaviors will also be common among these individuals. In addition to this, physical inactivity is commonly associated with the stress that comes from financial difficulty, limiting the time that the individual must exercise or reducing the motivation for the individual to exercise (8). All these behaviors increase an individual's likelihood of developing hypertension.

### Impact of debt and financial insecurity

It has been established through various studies that people who have a high amount of personal debt tend to suffer from elevated blood pressure. Having credit card debt, student loans and unpaid bills can contribute to the feeling of being financially burdened on a continuous basis and cause chronic anxiety and mental strain daily (9). Job instability and irregular income also contribute to this by creating an uncertain environment for the future. In addition, research has shown that someone who views their financial position as unstable is at a greater risk for both acute and chronic hypertension than someone in a stable financial position.

The best solution to solve stress is taking control in a clear step-by-step way. First gather all your credit cards, loans and bills. Note the balance, minimum payments and interest rates for each one. Check your bank and credit card statements to see where your money goes each month. To create a working budget, make a plan that balances your income and expenses. Give every dollar of your income a job like paying bills, saving or paying debt before the month starts. This way you know where your money is going. Cut back on essential spending like unused subscriptions eating out and impulse buying. If you need help, contact non-profit credit counseling agencies. They can help you combine debt or get interest rates. Also talk to a trusted advisor, for long-term planning and retirement goals. They can help you plan to achieve stability. Taking control of your finances takes time and effort. It is worth it. You can reduce stress and build a secure future.

### Sleep disturbances and their role in hypertension

Financial strain can lead to a wide range of mental and physical health issues, including insomnia and sleep disturbances. People who are worried about paying their bills often find it challenging to get enough sleep at night. When someone does not have enough sleep (which disrupts their body's normal circadian rhythm) and has an increased activation of the sympathetic nervous system, their blood pressure will increase. Research has shown that people who sleep < 6 h each night are more than twice as likely to have high blood pressure when compared with individuals who sleep at least 7 h each night, making sleep an essential factor in managing blood pressure levels (10).

There is a direct relationship between sleep problems and high blood pressure. Prolonged lack of sleep and sleep disorders like sleep apnea do not allow for the decrease in blood pressure during sleep at night. The continuous pressure on the vessels results in activation of the sympathetic nervous system, causing high blood pressure and cardiovascular diseases. Lack of sleep increases stress hormones, such as cortisol, putting the body in a state of “fight or flight,” causing higher blood pressure and heartbeat. Insomnia changes appetite-related hormones, increasing ghrelin levels and reducing leptin, causing obesity and diabetes.

### Socioeconomic status and health inequalities

The socioeconomic standing of people has a big impact on how financial mismanagement leads to hypertension (high blood pressure). It has been found that lower-income individuals are the most affected since they often have fewer resources (including financial) available to them, as well as being limited regarding the amount and quality of education and healthcare. It is possible that the financial mismanagement of lower income populations comes from social structures that create barriers to financial decision-making (such as holding down jobs that pay low wages but involve a very high cost of living). While numerous epidemiologic studies have documented higher rates of diagnosing hypertension among poorer populations, there is no question that when identifying the factors that impact health, socioeconomic factors are among the most important (11).

### Limited access to healthcare and preventive services

Individuals who experience financial hardships often have challenges accessing needed healthcare services such as routine visits, diagnostic tests, and antihypertensive prescriptions. Financial limitations can lead to individuals not being able to afford to see a physician within the appropriate time frame of their diagnosis, which creates the opportunity for a more advanced state of hypertension to develop because they do not have regular access to hypertension management or early diagnosis of hypertension. Preventive measures such as regular blood pressure checks, nutritional counseling, and stress management programs can also be difficult for individuals to access (12). This situation creates an environment for the continued progression of untreated hypertension and the associated complications (e.g., heart disease, stroke).

### Psychological factors and emotional stress

There is a significant link between financial problems and mental health issues like depression, anxiety, and feelings of helplessness. Mental health has caused a correlation between the two and is therefore likely to be responsible for rising heart rates, increased cortisol (a stress hormone), and the use of harmful coping strategies (13). Because of chronic worry about financial problems, individuals may develop a continuous cycle of stress since they are always ‘on guard' about future financial instability. The ongoing psychological burden has a profound effect on overall mental wellness and on how well the heart functions.

### Cultural and environmental influences

Social pressure and expectations of one's culture increase the negative effects of financial mismanagement. Many cultures expect an individual to fulfill their financial obligation to their social network or family; these pressures may lead to excess spending and accumulating debt. Environmental factors, including urban living, high costs of living, and inflation, add to the financial burden on individuals who live under these conditions (14). In addition, these external factors increase stress and create a higher probability of developing health problems, such as hypertension, in areas where there is a high-density population and there is a lack of economic opportunity.

### Evidence from epidemiological studies

Multiple population-based research projects have found that there is a link between economic hardship and hypertension. As an illustration, there are many documented cases of chronic economic stress associated with high reported levels of stress (15). Surveys have historically shown individuals who report chronic economic stress tend to be at an increased risk for developing hypertension; therefore, indicating a link between financial and physical health.

Financial problems happen when people or organizations do not manage their money well (15). This happens because of budgeting, lack of control over cash flow and unchecked problems with behavior or systems. Operating without a financial plan causes overspending and misallocation of funds. This happens when individuals or organizations do not plan their finances properly. They end up spending more than they should. Not saving for events, such as medical emergencies, job loss or economic downturns, forces individuals or businesses to rely on high-interest debt. This is a problem because it can lead to more financial troubles. Some people spend money than they earn. This is a recipe for disaster. They accumulate debt without a plan to pay it back. This can lead to ruin. Some problems, like addiction to gambling, substance abuse or impulsive spending can derail stability.

Hiding debts or spending habits from a partner or business associate can lead to financial ruin. Not understanding financial concepts like how interest works, tax obligations or investment risks is also a problem.

For businesses not separating duties, not doing audits, or not tracking expenses allows for silent losses or internal fraud. Over-expanding or investing much before having proper revenue streams or financial safeguards in place is also a problem. Retaining management or making poor strategic decisions such as bad mergers or unsuccessful marketing can also cause financial problems. These are some of the reasons why financial mismanagement occurs. It is a problem that needs to be addressed. Financial planning and management are crucial, for success. Individuals and organizations must take control of their finances.

## Conclusion

The issue of hypertension as it relates to poor money management represents a complex interplay of economic, psychological, and physiological factors. Many people with chronic financial related stress are very likely to develop hypertension because of how this chronic stress or financial stress can affect the body and because of the unhealthy coping behaviors that many use to deal with this chronic stress. The impact of this stress is heightened when we consider how many low-income people do not have adequate access to appropriate health care services. A multi-disciplinary approach to addressing this problem requires improving financial literacy among all segments of society, as well as creating environments that encourage healthy lifestyle choices and access to affordable health care services. Understanding the link between financial well-being and health can help policymakers and health care practitioners work cooperatively to develop holistic approaches to both preventing and managing hypertension, thereby improving the quality of life and decreasing the global burden of cardiovascular disease.
